# Woman’s Rights

**Published:** 1869-10

**Authors:** 


### WOMAN'S RIGHTS.

Woman has many rights, among which is that of being sick and miserable.

The right mentioned, seems to be the one most exercised by her.

This fact is startling, doubtless, but, nevertheless true.

And this is our argument:—

There is a constant strife among American women to present themselves to the public in a style, far exceeding their means—to "keep up appearances," as they term it. They have a perfect terror of losing *caste* in society, and will drudge and toil, early and late, and in secret, in order to secure the necessary means to enable them to display themselves at church, or at the concert, in the very latest styles of the dressmaker and milliner.

She has a choice of work, too, for be it known, there is only a certain class of employment that is "respectable"—so have they ordained.

To be a milliner, dressmaker, or tailoress, is highly honorable; but to be employed in cooking, washing and ironing, in a lady's kitchen, and for three times the wages, too, is degrading and entirely beneath the dignity of an American woman.

Hence have we thousands of miserable, sickly women, who stitch, stitch their lives away, in illy ventilated apartments, and by the aid of dimly burning lamps, in the vain attempt at deceiving the public into thinking that they are abundantly provided for.

We have frequently had them tell us that they were compelled to work until two or three o'clock in the morning, in order to complete the resquite amount of sewing to entitle them to sufficient wages to afford them the necessary food, light and fuel, for the following day and night. This constant use of the eyes upon black fabrics, and by artificial light, soon impairs the vision, and incapacitates them for their trade; while the confinement in badly ventilated apartments, (they can usually afford no other,) is certain destruction to health, sowing seeds of disease, from which they never recover. In this condition they consult the physician for the relief of their malady—he, of course, recommends other employment, exercise in the open air, better nourishment, etc. But, "what can I do?" exclaims the horrified invalid! "There is nothing else I can work at!"

Isn't there?

That is just what we purpose lecturing to you upon—you unfortunate creature. Let us see! You are weak, debilitated, and nearly blind. You require light, air, exercise and good food. Now, we will tell you where they all can be had, and within your means, too—

In the kitchen!

Go, seek employment in some respectable man's family—as domestic, nurse, housekeeper, and the like. But never sew. Of course, you will not be able to work hard at first, but you can probably peel potatoes, wash dishes, scour the knives or sweep a room, and so get up an appetite for your dinner—your medicine—precisely such as any sensible physician would prescribe for you.

You should be willing to work for this "medicine" alone, until returning health and strength entitle you to wages. When that time arrives, take charge of the kitchen—prepare the food—keep things cleanly and in order—be cheerful; work as though the house belonged to you, economize and plan for the best interest of your employer. You will gain health, friends, a home, respect, thanks and wages! In short, work like a Christian woman, at employment exactly suited to you—in a cheerful house—with a pleasant family—for good wages—and better health. You will not only gain the love and respect of all sensible people, but will better please yourself and your Maker.

You argue it isn't "respectable" to work as domestic. Isn't it, though? How unfortunate!

Do you retain the respect of your friends and acquaintances while you are toiling your life away, earning only a miserable existence as a seamstress? If so, they will think none the less of you for engaging in other honorable employment. If the domestic's life is not a "respectable" one, then make it so. If you enter the list, others will soon follow, and bless you for the example. Throw away this abominable thought of *caste* and engage in more honorable, healthful and useful employment. Throw down the needle and take up the frying-pan—go into the kitchen, and engage in the work that is suited to and expected of you. Do not be afraid of losing *caste* in community—better by far loose that, than health and happiness. Thousands of housewives will welcome you, pay you well, and bless you for your assistance. Our kitchens are full of blundering, ignorant Irish girls, many of whom fail to recognize the difference between a boot-jack and a grid-iron—release us from them, we pray you!

If the poor American girl, who has been taught household duties in her mother's family, would but come to the relief of those who are constantly annoyed by incompetent pretenders to the culinary art, the standard of domestic labor would soon rise far above that of the needle-woman, and all parties would be healthier, happier, and far better natured; and if the various woman's journals of the land would urge upon the sex to employ themselves more about our kitchens, and cease troubling themselves over politics and the right to vote, it would be far better for the poor, sickly ones about us, and more in accordance with our ideas of woman's rights.

